# Risk and Protective Factors Associated with Student Distress and School Dropout: A Comparison between the Perspectives of Preadolescents, Parents, and Teachers

**DOI:** 10.3390/ijerph191912589

**Published:** 2022-10-02

**Authors:** Maria Luisa Pedditzi, Roberta Fadda, Loredana Lucarelli

**Affiliations:** Department of Pedagogy, Psychology and Philosophy, University of Cagliari, Via Is Mirrionis 1, 09123 Cagliari, Italy

**Keywords:** parent–adolescent relationships, adolescent growth, protective and risk factors, student distress, school dropout, students–teacher interaction

## Abstract

This study investigates the principal risk and protective factors associated with student distress and school dropout in a group of preadolescents, through a qualitative and quantitative comparison between the perspectives of students, parents, and teachers. We administered a questionnaire to evaluate student distress and school dropout in a middle school (student’s age range 13–15 years). We afterwards analyzed the responses of students at risk of school dropout. We also administered a semi-structured interview with their parents and their teachers. The results indicated significant levels of school distress in students (Collective Distress > 8.75) and a problematic relationship between parents and students (22.42%). We also found a problematic relationship between teachers and students (24.39%). The main protective factor of student distress and school drop-out indicated by the students was a more sensitive and supportive relationship with both parents and teachers. On the contrary, parents and teachers indicated as possible causes of school distress and drop-out the intrinsic students’ motivation or other external factors associated with the community. The results of our study highlight the importance to apply a multidimensional and transactional psycho-educational model, in which the relationship between the students and their caretaker plays a major role.

## 1. Introduction

School dropout is a risk behavior that is a threat to individual wellbeing. School dropout concerns all the students that interrupt their school career without graduating. School dropout might also concern the students that, even though they go to school, do not react positively and in an adaptive way to the requests of the school. School dropout is often preceded by a period of psychological dropout, during which the students attend to the class, but they are emotionally detached [1].

Mancini and Gabrielli [2], who developed an important tool to measure the degree of school uneasiness, defined school uneasiness as an emotional state that might be expressed by a variety of behaviors such as low participation in school activities, low attention, school rejection, disruptive behaviors, problematic relationships with peers and teachers and low academic achievement. These difficulties do not allow the students to adequately experience the activities at school and to successfully learn at school, by using the maximum level of their cognitive, affective, and relational abilities. These difficulties in successfully facing the developmental tasks at school might bring some students to become disengaged for a prolonged period, and even to drop out of school.

School dropout is a dynamic process that starts well before the children will go to primary school [3,4,5]. It involves a series of demographic, social–cultural, familial, school, and individual factors: the home environment, the quality of early care, socio-economic status, the intellectual quotient, challenging behaviors, school achievements, peer relationships, and parental involvement [6].

From this perspective, school distress might be considered the first stage of a possible pathway, during which the concurrent action of risk factors and the lack of adult monitoring might expose the students to a maladaptive condition, toward isolation and possible antisocial behaviors [7].

### 1.1. Risk and Protective Factors in Family

School completion and dropping out of school are developmental processes with strong social and emotional antecedents. The early development of attachment bonds, and subsequent positive and negative behaviors and relationships set an early path toward school completion or drop out.

Emotional bonds to parents affect peer and teacher relationships and the school setting, affecting academic progress [8]. A robust literature on child-rearing practices indicates that attachment dynamics and early care substantially contribute to dropout and graduation processes [9].

The quality of the home environment and the quality of parenting are predictors of educational achievement and well-being.

The research results on authoritarian, permissive, and authoritative parenting styles [10] in the context of adolescents indicate that both authoritarian and permissive parenting styles were negatively associated with grades, and authoritative parenting was positively associated with academic success [11]. Parenting styles also relate to the way adolescents develop academic self-efficacy and attachments to their peers.

Lorca, Richaud and Malonda [12] indicated that parenting styles relate to the way adolescents develop attachments to their peers and to academic self-efficacy.

The permissive style is an important predictor of aggressive behavior and a negative predictor of attachment to their peers.

The research findings on parental social support also indicate that perceived parental support significantly predicts academic achievement and grade point average [13]. At-risk students, on the other hand, often cannot rely on their parents, and it is very difficult to communicate, especially in families characterized by conflicting relationships [14]. It is also important to consider the transitional period of adolescent development and the many changes in dynamics with parents. In the transactional model [3], the development is a product of the continuous dynamic interactions of the child and the experience provided by his or her social settings [4,5,6].

What is core to the transactional model is the analytic emphasis placed on the bidirectional, interdependent effects of the child and environment [3]. In a transactional model, parenting and child temperament are expected to mutually shape each other over time.

In the transactional relations, parents’ efforts might target reducing a child’s or adolescent’s negative affect and dysregulated behaviors. At the same time, it is these specific behaviors that might elicit more negative parenting that enhances behavioral and emotional problems [15]. Moreover, during infancy and adolescence, the balance between individual characteristics and the environmental demands tends to be constantly modified thanks to the emergence of various developmental tasks, which require new competences [3]. If there is a discrepancy between the needs of the individual and environmental requests (particularly from school and family), dysregulations or conflicts appear leading to various forms of risk and possible psychopathologic developments [4,5,6].

Battin-Pearson, Newcomb, Abbott, Hill, Catalano, and Hawkins [16] and Rumberger [17] associate the low expectations of parents related to academic achievements to school dropout in adolescents. Considering the relationship between students and parents, possible risk factors of school drop-out might be low involvement in school activities, inadequate supervision for homework, and low parental monitoring [18]. Parental monitoring is an important protective factor in adolescence against challenging behaviors in adolescents. It has been described as all the parental behaviors aimed to monitor the actions and behaviors of adolescents [18]. Recently, the studies considering parental monitoring shift their focus from the active and unidirectional role of parents in promoting wellbeing toward a dynamic and transactional approach, which considers the parent–son dyad [19].

Stattin and Kerr [20] highlighted the active role of adolescents in spontaneously informing their parents about their life during their spare time and outside the house. In the transition from childhood to adolescence, there is a reduction of the spontaneous information from the sons and the daughters and an increase in secrecy [20]. Moreover, an increase in secrecy in adolescents might predict norm violation and crime [21]. This might hamper the possibility to prevent and to intervening in reducing the risk of internalizing or externalizing behaviors.

### 1.2. Risk and Protective Factors at School

The concept of transactional relations is derived from an ecological perspective on development. Recent, interesting research findings show how an ecological model of family and classmate microsystems can help provide a comprehensive account of children’s academic achievement.

Several studies highlight that the quality of the student–teacher relationship varies according to the level of “goodness of fit” between the perceptions, the characteristics, the behaviors of the students, and the expectations of the teachers [22,23,24]. The teachers’ characteristics [25,26] might impact the educational relationship, particularly when considering the management of the behavior in the classroom, the teaching strategies, and the perception and the acceptance of the behaviors that do not match with the expectations [23,27].

The teachers that focus on punishment might increase the risk of dropout, and several studies indicate that the students who have those negative experiences at school might be more likely to dropout [28]. The students that drop out of school perceive the teachers as authoritarian, non-supportive, and non-interested in them [29], by perceiving the school as a place of boredom and in which they are not mirrored [6].

A study by Wang and Fredricks [30] highlighted that a better school adjustment might increase the willingness of the students to be more involved emotively and cognitively in school activities. This is an important protective factor for possible risk and criminal behaviors. In synthesis, it is important to adopt at school a developmental approach, to better understand school distress and adolescents’ problems, especially in presence of risk behaviors [31].

Recent studies also indicated the importance to adopt a multidimensional and transactional psychoeducational model to develop new interventions, aimed to prevent school distress and school dropout.

### 1.3. Aims of This Study

Several studies investigated the quality of parent–child and teacher–student relationships as possible risk and protective factors in school distress in adolescents.

However, few studies that considered the parental care [6] and the students–teachers relationship [22] as possible risk factors for school dropout considered the perspectives of students, teachers, and parents.

We are therefore interested in whether, from the perspective of at-risk preadolescents, the relationship with their parents and teachers can be considered a risk factor and whether it can also be shared by parents and teachers.

In a sample of preadolescents, this study, therefore, aimed to:

(1) Investigate the degree of school distress of preadolescent students attending a middle school.
(2) Investigate the principal risk factors that emerge from the perspective of the students, teachers, and parents.
(3) Verify whether the quality of parent–student and teacher–student relationships are considered by parents, teachers, and students as a possible protective factor.
(4) A qualitative comparison between the student’s perspective, parental perspective, and teacher’s perspective.

## 2. Materials and Methods

To investigate school distress and school dropout, we administered a questionnaire on school dropout to students at a middle school. We next interviewed parents and teachers of students at risk of dropping out of school, and afterwards we analyzed the content of the interviews with the software SPAD—Système Portable Pour l’Analyse des Données Textuelles [32]. Data collection was done before the COVID-19 pandemic. The study was approved by the Ethical Committee of the University of Cagliari. All the students’ parents signed the written consent form to participate in the study.

### 2.1. Participants

In the first phase of the research, 120 preadolescents attending a middle school (55% male and 45% female) between the ages of 11 and 15 participated in the survey. A qualitative study then focused on students at risk of school dropout (22.5%) to investigate risk and protective factors from the perspective of their parents and teachers. At-risk preadolescents were 22.5% of the sample (62.1% males; 37.9% females), aged range 13–15 years. The parents who participated in the study (75% mothers and the 25% fathers) ranged in age from 30 to 58 years, with a mean age of 44 years. The teachers were from classes in which the risk of dropout was present (75% females and 25% males). Data collection took place in a suburban area of Cagliari (Italy), where the risks of school drop-out, psychological and social malaise, drug abuse, and unemployment were high [33].

### 2.2. Instruments

The questionnaire TVD—the school dropout test by Mancini and Gabrielli [2], allows for analyzing the school distress of the students across various dimensions: the concept of self and the relationship with the school, the teachers, the peers, and the parents. Here are some examples of the items:

- “I talk about the school with my parents…”.
- “I need that my parents would…”.
- “Sometimes my teachers…”.
- “If I was a teacher…”.

The coding of the TVD [2] allows us to categorize the single answers into positive, negative, and neutral, and to attribute the relative scores. The analysis of the negative items will allow us to compute an index, called the Individual Distress (I.D.), as follows:

- I.D. = 10–11 signs of distress.
- I.D. = 12–13 moderate grade.
- I.D. = 14–21 severe grade.

It is also possible to calculate another index, the Collective Distress (Collective Distress), by dividing the number of negative answers by the total number of students per class (C. D. = N/n). If the C.D. is above 8.75 (cut-off for the population considered), it is possible to compute the severity of distress. It is also possible to calculate the percentage of distress in relation to the following dimensions:

- The concept of self.
- Student–parent relationship.
- Teacher–parent relationship.
- Student–peers relationship.
- The relationship with the school.

For the psychometric properties of the TVD, please see Montanari [34]. Parents and teachers have been interviewed with the following open-ended questions:

“Referring to your students/children”:

1. “What are the possible causes of school dropout?” (Both, parents and teachers).
2. “How could the school reduce school dropouts?” (Teachers).
3. “How could the family reduce school dropouts?” (Parents).

### 2.3. Analysis of the Interviews

The parents’ and teachers’ answers to the interviews were analyzed [35] with the software SPAD—Système Portable Pour l’Analyse des Données Textuelles [32].

This software analyzed the lexicon used in a written text by considering the single sentence as a unit. The analysis includes the following steps:

1. Development of a vocabulary from a synthesis of the interviews.
2. Analysis of a vocabulary by excluding the articles, the prepositions, the conjunctions and by excluding the “empty words” (cancelled).
3. Identification of the most representative words according to the frequency and by disambiguating the terms with a variety of meanings.
4. Development of the categories of meaning and merging of the words with the same meaning or which are equivalent.
5. Use of specific categories of analysis or macro-categories (i.e., causes, risk factors) and micro-categories (i.e., school, family).

Finally, we analyzed the specific words (Key Words in Context), so that we could identify the term most used by the adults, their frequency, and the likelihood of use in each subcategory (teachers and parents). The level of significance was *p* ≤ 0.01.

## 3. Results

### 3.1. School Distress in Students

The analysis conducted on the total sample of students (*n* = 120) showed collective distress above the risk cut-off (DC/N > 8.75) in one group of students (22.5% of the sample). The group consisted of 27 preadolescents (62.1 percent male; 37.9 percent female) in the 13–15 age group.

Specifically, we found the following distribution in the areas of distress (test TVD) [2]: student–teacher relationship (24.39%), parents–students relationship (22.42%), relationship with peers (18.65%), self-concept (17.32%), relationship with the school (17.22%). As it is possible to see, the most common areas of distress were the relationship with parents and teachers. Then, we qualitatively analyzed the single answers given by the students about their relationships with teachers and parents.

### 3.2. Students-Parents Relationship

Here, we analyzed the following questions in the school dropout test—TVD Test.

- “When I talk with my parents about the school”:
  - “I get bored” (16.67%).
  - “I avoid this topic” (16.67%).
  - “My parents bother me” (16.67%).
  - “My parents get me nervous” (11.11%).
  - “My parents tell me to study” (11.11%).
  - “I walk away” (11.11%).
  - “I go to bed” (5.55%).
  - “I walk away otherwise they overwhelm me with too many questions” (5.55%).
  - “I pretend that I’m not listening” (5.55%).

The most frequent answers given by the students were related to negative states raised by the possible mediating role of the parents with the school tasks, such as getting bored or avoiding talking about the school, or getting irritated (χ^2^ = 16.69; df = 8; *p* < 0.05).

Interactions between preadolescents and their parents can be analyzed by considering the transactional dynamics between parents and children in the delicate phase of transition to adolescence.

The students use active communicative strategies to modify the transitions with their parents and to protect their privacy and their autonomy (“I pretend to listen”, “I go to bed”). The parents’ attempts to “elicit” the information are labelled as negative by the students (“My parents bother me”, “My parents get me nervous”).

An analysis of the responses given by at-risk preadolescents reveals the presence of avoidance behavior toward their parents, who probably have difficulty relating to them at this delicate stage of transition to adolescence.

For the second question, the students answered as follows.

- “I need that my parents”:
  - “Help me” (31.7%).
  - “Understand me” (15.8%).
  - “Help me this year with the final exam” (10.5%).
  - “Don’t push me too hard” (10.5%).
  - “Will be less demanding” (10.5%).
  - “I could trust more in our relationship” (5.25%).
  - “Give me more money” (5.25%).
  - “I don’t need anything from my parents. I’m independent” (5.25%).
  - “We are happy as we are. I won’t change anything” (5.25%).
  - Other (5.25%).

The students’ answers indicate their need to be “helped” and to be “understood”. Moreover, they would like for their parents to be less “demanding” and “more understanding” (χ^2^ = 52.59; df = 8; *p* < 0.05).

Other answers indicate a discrepancy between the needs of the students and the parental expectations (“I would like that my parents would be less demanding”, “I would like that my parents won’t push me that much”).

Students express several needs about the relationship with their parents (“I need help from my parents”, “I need that my parents understand me”, “I need to have a relationship in which I can trust in my parents”, and “I need more money from my parents”).

The teens interviewed describe their parents’ parenting styles as authoritarian, and a need for a more authoritative relationship with their parents seems to emerge in them. Only 5.25% of the answers indicated that the parents “Are good as they are”.

### 3.3. The Relationship between Students and Teachers

Here, we indicated the most frequent answers given by the students about the relationship with the teachers.

- “Sometimes my teachers…” (class A):
  - “Are unpleasant” (25%).
  - “They bother me” (25%).
  - “They don’t understand me” (25%).
  - “They are pleasant but only sometimes” (8.33%).
  - “They don’t understand that it is not always the student’s fault” (8.33%).
  - “They punish without a reason” (8.33%).
- “Sometimes my teachers…” (class B):
  - “Are very strict” (30.8%).
  - “They bother me” (23%).
  - “They make me bored” (15.4%).
  - “They help me” (15.4%).
  - “They get upset because we don’t study” (7.7%).
  - “They are firm” (7.7%).

In class A, the students describe the teachers using negative terms (“They are unpleasant”, “They bother me”, and “They are incapable to understand the real needs of the students”).

In class B, the students underly the teachers’ strictness and the fact the teachers frequently become upset. The students described the desired characteristics of the teachers as follows:

- “If I was the teacher…”:
  - “I would try to render the lesson more interesting” (12.5%).
  - “I would help the students” (12.5%).
  - “I won’t give too much homework” (12.5%).
  - “I would be less strict” (12.5%).
  - “I would socialize more with the students” (12.5%).
  - “I would try to understand the students and to talk with them” (12.5%).
  - “I wouldn’t give too much homework” (6.25%).
  - “I would organize a party with my students” (6.25%).
  - “I would have fun in punishing the students” (6.25%).
  - “I would never be a teacher” (6.25%).

The term “funny” is present several times in describing the desired lectures, as well as the term “help” and “understanding”. Once again, it emerges from the student’s perspective that they need a more human, affective, and funny relationship with the teachers. In general, the perceived quality of the students–teachers’ relationship is poor [23].

The students describe their relationship with their teachers as authoritarian in nature.

As already indicated in reference to their parents, the at-risk adolescents considered seem to desire instead a more authoritative type of relationship with teachers as well.

### 3.4. The Causes of School Dropout for the Teachers

Referring to the classes at risk of school dropout that emerged from the survey and then referring to the relationship with students in those specific classes, the teachers answered to the first question “What is, in your opinion, the cause of school dropout?” with the following terms (Table 1).

These terms are the most significantly present in the text with a level of probability of *p* ≤ 0.01.

The words most used by the teachers to answer to the question about school drop-out are the following: environment (*p* = 0.000), missing (*p* = 0.002), motivation (*p* = 0.003); and family (*p* = 0.006).

The words significantly less used by the teachers are the following, as indicated in Table 2 (*p* ≤ 0.01).

The words “students” and “teachers” are less used by the teachers (*p* ≤ 0.01) (Table 2).

Here are some of the teachers’ answers to the question: “What are, according to your opinion, the most significant causes of school drop-out?”:

- “School dropout is due mainly to the inability of the family to motivate the students and to monitor them”.
- “It is mainly due to the lack of motivation of the students, their low level of expectations and the problems with the family”.
- “In my opinion, the cause is the students’ familiar distress, which intensifies the school distress. School dropout is due to motivation and socio-cultural background”.

Teachers tend to attribute the causes of the school dropout mainly to external factors, like family, and to the student’s motivation. Terms like “student” and “teachers” are absent, as the teachers do not consider their responsibility in school dropout.

### 3.5. The Parent’s Perception of the Causes of School Dropout

Parents answered as follows (Table 3) to the first question “What are, in your opinion, the causes of school dropout in this school?”, (*p* ≤ 0.01).

The words most used by the parents to explain the possible causes of students drop-out in preadolescents at risk are the following: “school” (*p* = 0.000). “disinterest” (*p* = 0.001), “answer” (*p* = 0.002), community (*p* = 0.003), friendship (*p* = 0.005), and services (*p* = 0.007).

Then, we considered the parents’ most absent words in the identification of the possible causes of students’ drop-out, as indicated in Table 4 (*p* ≤ 0.01).

The words “sons/daughters” and “relationship” are the most significantly absent (*p* < 0.01) in the texts of the parents’ interview (Table 4).

Here are some answers that parents of students at risk gave to the question “According to your opinion, what are the possible causes of school drop-out?”:

- “School dropout is surely related with the student-teacher relationship, since teachers are often not interested in the students”.
- “Students are poorly motivated to study. Moreover, they have bad friends which lead them to leave the school”.
- “A possible cause of school dropout is the lack of services in the community, like sports activities, which hampers the possibility to find good friendship”.
- “ Teenagers don’t like to talk about their school problems with their parents”.

Parents consider the school and the community unable to answer to the students’ needs. Terms like “son/daughter” (*p* = 0.000) and “relationship” (*p* = 0.001) are absents.

These terms are probably perceived as the closest to their role as parents and, therefore, are excluded by the risk factors considered.

The difficulties in communicating with children emerge in the last response, given by some parents.

They seem to share the same difficulties in communicating already expressed by preadolescents.

They, however, remain vague and prefer not to elaborate on their difficulties with their children.

### 3.6. Protective Factors according to Parents

Parents in classes at risk of school dropout were asked questions whose delivery was as follows.

Referring to your son/daughter, we ask you to answer the following question: “How the family might prevent school dropout?” (Table 5).

The terms are present in a statistically significant way in the text (*p* ≤ 0.01).

The most frequent words are: “students” (*p* = 0.000), “relationship” (*p* = 0.000), “adult” (*p* = 0.000), and “communication” (*p* = 0.007).

The words less used by the parents are the following (Table 6):

The words significantly less used by the parents are “others” (*p* = 0.000), “studying” (*p* = 0.002), “school” (*p* = 0.005), and “dropout” (*p* = 0.005).

The term “studying” is significantly absent from the protective factors, as the parents do not attribute the responsibility of their disengagement to the student.

Here are some most typical answers that the parents gave to the following question “How could the family reduce school dropout?”:

- “By creating a good relationship with students, despite it is difficult to communicate with them”.
- “It is important to communicate with the students’ event thought they isolate themselves and they think that we cannot understand them”.
- “Trying to understand their need to communicate even though they do not express it”.

According to the parents, the quality of the communication between adults and students is a possible cause of the students’ distress.

### 3.7. Protective Factors according to the Teachers

At the question: “How the school could reduce school dropouts?”, the teachers indicate the following terms (Table 7).

The words most used by the teachers are: “useful” (*p* = 0.000), “education” (*p* = 0.001), “learning” (*p* = 0.002) and “recover” (*p* = 0.004).

The words less used by the teachers are in Table 8.

The terms “missed” (*p* = 0.001), “abandoned” (*p* = 0.004), and relationship (*p* = 0.010) are less frequent. Here are some teachers’ answers that are typical to indicate the protective factors at school:

- “To activate services of psychological support, to implement intervention programs to recover the lack of abilities in various disciplines and to educate the teachers”.
- “Implement students’ self-esteem and motivation, by recognizing the importance of what they know rather than focusing on the lack of knowledge”.
- “Surely to invest more on teacher education and to involve more the families”.

The teachers recognize the need to adopt intervention strategies to improve the communication between the students, the school, and the families.

## 4. Discussion

The research results show that within the school considered, 22.5% of students are at risk of dropping out of school.

The results of our study indicate in this group of at-risk students an age range of 13 to 15 years and high levels of school distress (C.D. > 8.75). We also found in preadolescents at risk of dropout a negative student–parent relationship (22.42%) and student–teacher relationship (24.39%), as perceived by the students. Considering the students’ answers to the TVD-test [2] about their relationship with their parents, the students show a high level of distress in talking about homework, so that they tend to avoid the topic or to discredit the importance of the topic.

The students manifest their need to be helped by their parents with conflictual and ambivalent behaviors. At the same time, the parents do not understand their responsibilities in determining the students’ distress.

They believe that the cause of distress is entirely determined by the school. On the contrary, the students consider help from their parents to be very important, even though they do not help the parents to understand their needs (“I walk away”, “I got bored”).

Both parents and students show several difficulties in communicating about the school, even though the parents understand the importance of good communication for a good relationship with their children.

Thus, among the protective factors that emerge in common among students, parents, and teachers is the need for a more authoritative relationship with each other. Regarding the relationship with teachers, students perceive the teachers in the TVD [2] as punitive, excessively strict, and incapable of understanding their affective needs.

There is a discrepancy between the teacher’s characteristics and the student’s needs, which would like to entertain new ways of communication, based on the highest levels of affectivity and understanding.

For parents and teachers, school dropout depends on external causes rather than their own specific roles. For the teachers, the roots of school dropout are in the family. For the teachers, school dropout depends on the family and on the students’ personal motivation. Thus, both parents and teachers tend to deny their own responsibility.

These results seem to indicate a possible contrast between school and family and certainly a lack of tools to prevent school dropout.

Few general parents’ responses seem to refer to their communication difficulties with preadolescents. These responses call for further investigation, aimed at better exploring their problems in communicating with their children. Such closure may be in line with a disengaged and avoidant parenting style. Further exploration would also clarify any and further obstacles to the interview.

## 5. Conclusions

The findings of this research confirm that the quality of the parent–child (9) and teacher–student (22) relationship are important risk and protective factors with respect to school dropout, and that parenting styles and parental monitoring can influence students’ perceptions of their relationship with their parents and teachers. Regarding parents and teachers, it emerges that they, too, believe that the relationship with students is an important protective factor, but it is observed that the causes of dispersal are assigned from the family to the school and vice versa from the school to the family without taking responsibility for the failure of educational action.

In line with the transactional perspective of Sameroff [3], we found that there is a discrepancy in the perspective of students, parents, and teachers regarding the expectations and the requests of both parents and teachers that might cause school drop-out. Thus, future intervention programs should focus on the quality of the students–caretakers relationship as a possible protective factor against school dropout.

New programs should be developed to include parent training, aimed to elaborate conflicts and to solve possible forms of dis-regulation in the students–parents relationship [5]. These results also suggest the importance of considering the relationship between students and teachers and that both parents and teachers should focus on their relationships with the students [36,37,38].

According to a meta-analysis by Lionetti et al. [18], which focuses on the parental monitoring, even though preadolescents tend to respond with secrecy to their parents’ questions about school, parental monitoring is an important protective factor for school distress and school dropout.

Our study has some limitations that need to be acknowledged. Our approach is qualitative. Thus, we cannot evaluate the strength of the influence of the risk factors considered. New research might be necessary to apply a quantitative approach, which could better support our results. Since this is an exploratory study, however, we aim to continue this research with the use of specific objective instruments to analyze educational practices and attachment dynamics to parents in preadolescence.

Moreover, our data collection was done before the COVID-19 pandemic. Another possible future line of research might be to address homogenous groups of preadolescents at risk of school dropout now, after the COVID-19 pandemic, to verify the impact of the pandemic on the perception of the risk and protective factors in this population. It might be of interest to verify whether parental monitoring has been affected by the pandemic.

To conclude, the results of our study demonstrate the importance to monitor the adaptive and emotional functioning of the students both in family and at school in preadolescents, from a multidimensional and transactional psychoeducational perspective. These models might successfully implement a new intervention program to prevent school distress and school dropout.

## Figures and Tables

**Table 1:** Teachers’ answers to the question: “What is, in your opinion, the cause of school drop-out?”.

Characteristics Terms and/or Sentences	% Internal	% Global	Internal Frequency	Global Frequency	Value of the Text	Probability
environment	1.98	0.21	16	16	3.718	0.000 ^1^
missing	1.95	0.84	12	24	2.890	0.002 ^1^
motivation	2.60	1.34	16	38	2.704	0.003 ^1^
family	2.60	1.41	16	40	2.497	0.006 ^1^
factor	1.98	0.62	9	12	1.913	0.018 ^2^
bounded	0.65	0.21	4	6	2.007	0.022 ^2^
time	0.49	0.14	3	4	1.827	0.034 ^2^
institution	0.49	0.14	3	4	1.827	0.034 ^2^
lacking	0.65	0.25	4	7	1.713	0.043 ^2^
questions	0.33	0.07	2	2	1.677	0.046 ^2^
ignorance	0.33	0.07	2	2	1.677	0.047 ^2^

^1^ Level of probability of *p* ≤ 0.01. ^2^ Level of probability of *p* ≤ 0.05.

**Table 2:** The words less used by the teachers in answering to the question: “What is, in your opinion, the cause of school drop-out?”.

Characteristics Terms and/or Sentences	% Internal	% Global	Internal Frequency	Global Frequency	Value of the Text	Probability
students	0.33	2.18	2	62	−3.955	0.000 ^1^
teacher	0.81	2.15	5	61	−2.623	0.004 ^1^
class	0.16	0.95	1	27	−2.276	0.011 ^2^
power	0.49	1.41	3	40	−2.151	0.016 ^2^
part	0.16	0.84	1	24	−2.022	0.022 ^2^
risk	0.00	0.42	0	12	−1.614	0.033 ^2^
school	0.00	0.39	0	11	−1.490	0.048 ^2^

^1^ Level of probability of *p* ≤ 0.01. ^2^ Level of probability of *p* ≤ 0.05.

**Table 3:** Parents’ answers to the question “What are, in your opinion, the causes of school dropout?”.

Characteristics Terms and/or Sentences	% Internal	% Global	Internal Frequency	Global Frequency	Value of the Text	Probability
school	2.64	1.41	12	22	2.487	0.000 ^1^
indifference	1.32	0.46	6	13	2.323	0.001 ^1^
answer	1.76	0.42	8	12	3.697	0.002 ^1^
community	0.66	0.11	3	3	2.648	0.003 ^1^
friendship	0.88	0.18	4	5	2.769	0.005 ^1^
services	0.44	0.07	2	2	1.952	0.007 ^1^
sport	0.44	0.07	2	2	1.952	0.028 ^2^
a lot	2.86	1.69	13	48	1.834	0.033 ^2^
finding	0.88	0.32	4	9	1.734	0.048 ^2^

^1^ Level of probability of *p* ≤ 0.01. ^2^ Level of probability of *p* ≤ 0.05.

**Table 4:** The words less used by the parents in answer to the question: “What is, in your opinion, the cause of school drop-out”.

Characteristics Terms and/or Sentences	% Internal	% Global	Internal Frequency	Global Frequency	Value of the Text	Probability
Sons/daughter	0.44	2.15	2	61	−2.916	0.000 ^1^
relationship	0.32	1.77	2	52	−2.217	0.001 ^1^
different	0.00	0.42	1	12	−1.158	0.013 ^2^
finding	0.00	0.42	1	12	−1.158	0.023 ^2^
relationship	0.22	0.77	1	22	−1.217	0.032 ^2^
believe	0.00	0.39	1	11	−1.050	0.047 ^2^

^1^ Level of probability of *p* ≤ 0.01. ^2^ Level of probability of *p* ≤ 0.05.

**Table 5:** Parents’ answers to the question “How the family might prevent school dropout?”.

Characteristics Terms and/or Sentences	% Internal	% Global	Internal Frequency	Global Frequency	Value of the Text	Probability
students	4.89	2.18	34	62	5.055	0.000 ^1^
relationship	2.44	0.77	17	22	5.022	0.000 ^1^
adults	4.17	2.15	29	61	3.829	0.000 ^1^
communication	0.86	0.32	6	9	2.371	0.007 ^1^
report	0.72	0.25	5	7	2.269	0.012 ^2^
risk	1.01	0.42	7	12	2.242	0.013 ^2^
establish	0.43	0.11	3	3	2.180	0.025 ^2^
person	1.01	0.46	7	13	2.025	0.031 ^2^
dialog	0.43	0.14	3	4	1.667	0.048 ^2^

^1^ Level of probability of *p* ≤ 0.01. ^2^ Level of probability of *p* ≤ 0.05.

**Table 6:** The words less used by the parents in answer to the question: “How the family might prevent school dropout?”.

Characteristics Terms and/or Sentences	% Internal	% Global	Internal Frequency	Global Frequency	Value of the Text	Probability
others	0.43	3.13	3	89	−5.357	0.000 ^1^
studying	0.14	1.06	1	30	−2.838	0.002 ^1^
school	0.43	1.41	3	40	−2.544	0.005 ^1^
dropout	0.00	0.67	1	19	−2.595	0.005 ^1^
social	0.43	1.34	3	38	−2.392	0.021 ^2^
family	0.00	0.42	1	12	−1.823	0.034 ^2^
often	0.14	0.53	1	15	−1.364	0.046 ^2^

^1^ level of probability of *p* ≤ 0.01. ^2^ level of probability of *p* ≤ 0.05.

**Table 7:** Teachers’ answers to the question “How the school could reduce school dropout?”.

Characteristics Terms and/or Sentences	% Internal	% Global	Internal Frequency	Global Frequency	Value of the Text	Probability
useful	0.69	0.18	12	19	3.077	0.000 ^1^
education	1.11	0.42	10	9	2.744	0.001 ^1^
learning	1.00	1.14	9	8	2.641	0.002 ^1^
recover	0.55	0.14	8	6	2.641	0.004 ^1^
intervention	0.69	0.25	5	3	2.205	0.024 ^2^
activity	0.42	0.11	3	3	2.136	0.036 ^2^
programs	0.55	0.18	2	4	2.132	0.046 ^2^

^1^ Level of probability of *p* ≤ 0.01. ^2^ Level of probability of *p* ≤ 0.05.

**Table 8:** The words significantly less used by the teachers in answering to the question: “How the school could reduce school dropouts?”.

Characteristics Terms and/or Sentences	% Internal	% Global	Internal Frequency	Global Frequency	Value of the Text	Probability
missed	0.00	0.84	0	24	−3.137	0.001 ^1^
abandoned	0.00	0.67	0	19	−2.674	0.004 ^1^
relationship	0.14	0.77	1	22	−2.220	0.010 ^1^
following	0.00	0.39	0	11	−1.756	0.020 ^2^
going	0.14	0.49	1	14	−1.312	0.035 ^2^
phenomenon	0.00	0.28	0	8	−1.307	0.046 ^2^

^1^ level of probability of *p* ≤ 0.01. ^2^ level of probability of *p* ≤ 0.05.

## Data Availability

Upon reasonable request, data used and analyzed during the current study are available from the corresponding author.
